# Development of root resorption during orthodontic tooth movement after cleft repair using different grafting materials in rats

**DOI:** 10.1007/s00784-022-04537-3

**Published:** 2022-05-14

**Authors:** Stephan Christian Möhlhenrich, Kristian Kniha, Zuzanna Magnuska, Sachin Chhatwani, Benita Hermanns-Sachweh, Felix Gremse, Frank Hölzle, Gholamreza Danesh, Ali Modabber

**Affiliations:** 1 Department of Orthodontics, University of Witten/Herdecke, Alfred-Herrhausen Str. 45, 58455 Witten, Germany; 2 Department of Oral and Maxillofacial Surgery, University Hospital of Aachen, Pauwelsstraße 30, 52074 Aachen, Germany; 3 Institute for Experimental Molecular Imaging, Department of Nanomedicine and Theragnostic, RWTH Aachen University, Forckenbeckstraße 55, 52074 Aachen, Germany; 4 Implant Pathology, ZBMT, Campus Melaten, Pauwelsstraße 17, 52074 Aachen, Germany

**Keywords:** Cleft repair, Jaw reconstruction, Grafting materials, Cleft palate, Root resorption, Tooth movement

## Abstract

**Objective:**

The aim of the present study was to investigate the influence of three grafting materials for cleft repair on orthodontic tooth movement in rats.

**Materials and methods:**

Artificial alveolar clefts were created in 21 Wistar rats and were repaired 4 weeks later using autografts, human xenografts and synthetic bone substitute (beta-tricalcium phosphate/hydroxyapatite [β-TCP/HA]). A further 4 weeks later, the first molar was moved into the reconstructed maxilla. Microfocus computed tomography (μCT) was performed six times (T0–T5) to assess the tooth movement and root resorption. After 8 weeks, the affected reconstructed jaw was resected for histopathological investigation.

**Results:**

Total distances reached ranged from 0.82 ± 0.72 mm (β-TCP/HA) to 0.67 ± 0.27 mm (autograft). The resorption was particularly determined at the mesiobuccal root. Descriptive tooth movement slowed and root resorption increased slightly. However, neither the radiological changes during tooth movement (µCT T1 vs. µCT T5: autograft 1.85 ± 0.39 mm^3^ vs. 2.38 ± 0.35 mm^3^, *p* = 0.30; human xenograft 1.75 ± 0.45 mm^3^ vs. 2.17 ± 0.26 mm^3^, *p* = 0.54; β-TCP/HA: 1.52 ± 0.42 mm^3^ vs. 1.88 ± 0.41 mm^3^, *p* = 0.60) nor the histological differences after tooth movement (human xenograft: 0.078 ± 0.05 mm^2^; β-TCP/HA: 0.067 ± 0.049 mm^2^; autograft: 0.048 ± 0.015 mm^2^) were statistically significant.

**Conclusion:**

The autografts, human xenografts or synthetic bone substitute used for cleft repair seem to have a similar effect on the subsequent orthodontic tooth movement and the associated root resorptions.

**Clinical relevance:**

Development of root resorptions seems to have a secondary role in choosing a suitable grafting material for cleft repair.

**Supplementary Information:**

The online version contains supplementary material available at 10.1007/s00784-022-04537-3.

## Introduction

For cleft repair, autologous bone grafts from different donor sites (e.g. iliac crest, cranium, tibia, rib and mandibular symphysis) and commercially available grafting materials such as allografts, xenografts and synthetic bone substitutes (e.g. bioceramics, polymers or biocomposites) can be used [1–3]. However, bony autografts, especially the grafts from the iliac crest, are considered the gold standard for cleft repair due to their osteogenic, osteoinductive and osteoconductive properties [4, 5]. Nevertheless, the grafting process has some operative risks and postoperative morbidities, including pain, haematoma and delayed ambulation. Furthermore, the region-specific limited bone supply and the inherent susceptibility to resorption in the long term must be taken into consideration [6–11]. Agents like recombinant human bone morphogenetic protein and demineralized bone matrix can also be used, but there may be potential for local or immune reactions, graft failure, infection and need for additional surgery [5]. Therefore, further investigations have been carried out to improve alternative bone substitute materials such as xenografts, ceramics, polymers or biocomposites in terms of having better clinical outcomes and reduced postoperative morbidity [3, 4, 12].

Occasionally, bone substitutes are also relevant in orthodontics. For example, these may be considered if orthodontic tooth movement across a narrow alveolar ridge area is necessary. Otherwise, this would inevitably entail some adverse reactions such as limited movement or periodontal tissue damage. In this context, in a current review by Lu et al., it was mentioned that the vast majority of studies have confirmed that teeth can be moved through bone defects augmented with bone grafts, despite slight occasional root resorptions [13]. The authors recommended that orthodontic tooth movement into such constructed alveolar ridge should be not initiated before 4 weeks after implantation.

Towards the aforementioned end, many different experimental cleft models in rats have been developed [14–22]. However, most of these models do not correspond to the clinical situation in terms of anatomical defect, which is clinically characterised by an epithelial-lining covering. Therefore, the cleft surface must be covered with healthy mucosa when cleft repair is to be performed. To do this, an artificial alveolar cleft must be generated in a two-stage surgery, whereby the mucosal lining of the cleft will be achieved after the healing period [23]. Also different from clinical practice are the previously introduced cleft regions: the mid-palate cleft in the anterior part of the maxilla [14–16] and the alveolar cleft in the central [15, 17, 18] or posterior [19–22] maxilla. With regard to their position and anatomy, only the posterior alveolar cleft allows subsequent tooth movement into the alveolar-cleft bone graft area. However, this bone deficit is usually based on the extraction of the first molar, which makes the defect more a large extraction defect than a complete interruption of the maxillary continuity in an alveolar cleft.

Actually, the influence of bone substitutes and their long-term outcomes in cleft models in combination with subsequent orthodontic tooth movement remain unclear. Sun et al. found that orthodontic movement into the reconstructed area can facilitate bone reconstruction through stimulation, which enhances the bone remodelling and provides a bone matrix for shifting teeth [20]. Ru et al. compared a bovine xenograft with a synthetic substitute mixture of beta-tricalcium phosphate (β-TCP) and hydroxyapatite (HA) in a related rat alveolar-defect model and reported the least amount of tooth movement and the lowest root resorption and crater volumes in the synthetic-bone-substitute group [21]. They supposed that bovine xenografts have less osteoconductive potential than the synthetic substitutes. In this context, Norten et al. reported that bovine bone substitutes in humans degrade slowly and could be responsible for uncertain immune responses and fibrous encapsulation with healing [24]. Allogeneic grafting materials are already in use for cleft repair and promise good results, such as decreased operation time, reduced hospital stay and less graft resorption over time [25–27].

No information has been given, however, about the influence of human grafting material as bone substitute on the subsequent orthodontic tooth movement in bony repaired clefts. As such, this study was conducted to determine root resorptions resulting from the use of three different underlying grafting materials (autografts, human xenografts and synthetic bone substitutes [β-TCP/HA]) and to compare these with each other. The study hypothesis was that human xenografts and β-TCP/HA bone substitute led to more root resorptions than autologous bone, which is currently the gold standard.

## Materials and methods

**Fig. 1:**
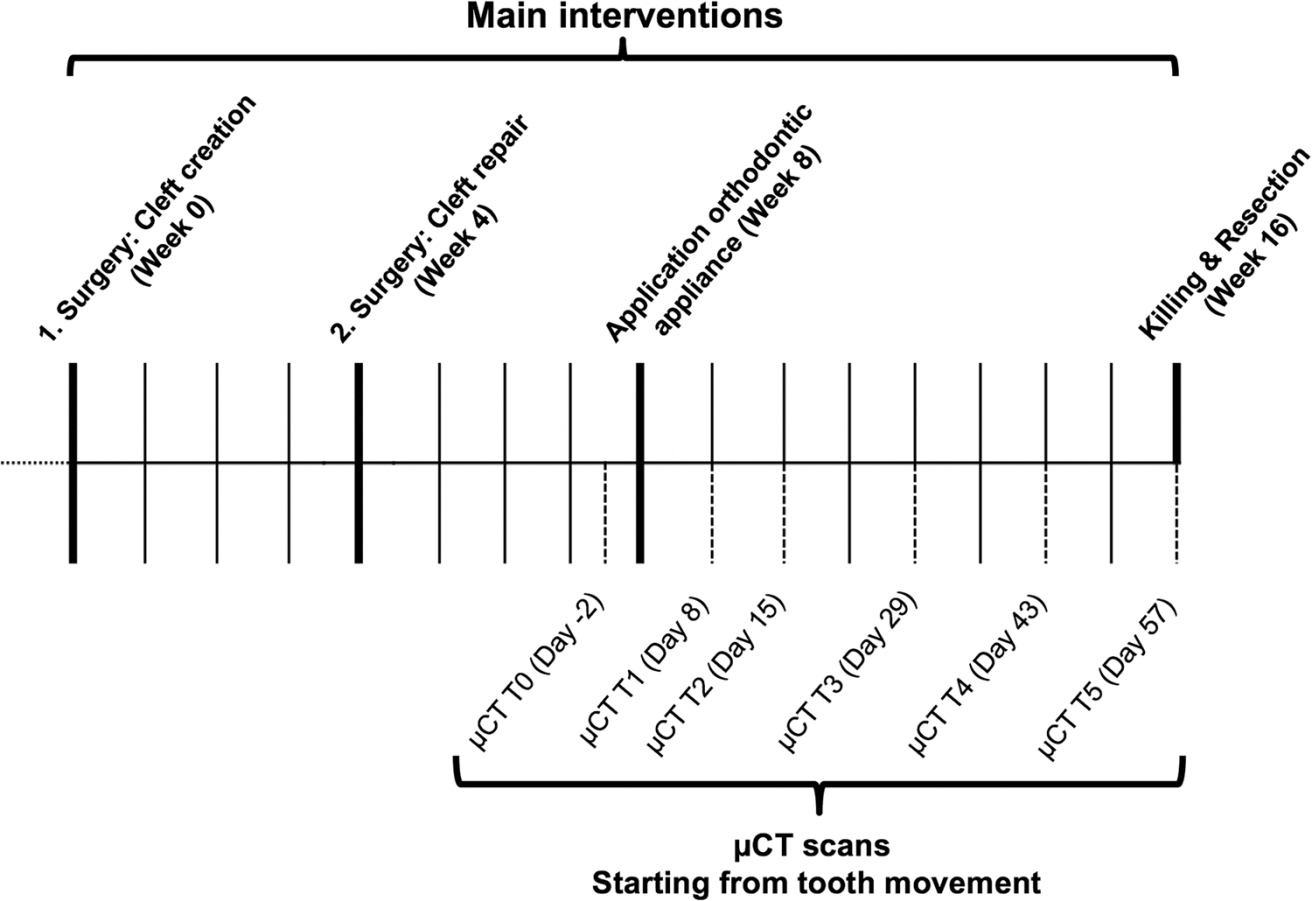
Timeline of the research protocol. The thick, solid lines represent the main experimental interventions under intraperitoneal injection: cleft creation, cleft repair, application of orthodontic appliances, sacrifice and resection. The dotted lines represent the radiological measures in microfocus computed tomography for monitoring under isoflurane anaesthesia

The a priori sample size calculation was based on the data previously published by Ru et al. for mean apical root resorption in rats treated with xenogeneic/bovine and synthetic β-TCP/HA bone [21]. The calculation was achieved using one-way analysis of variance (ANOVA) with regard to the root resorptions. The sample size estimation relied on the large observed effect (0.0605 vs. 0.089) and the related difference between xenogeneic and autologous bone graft, which was expected to be half the difference between the xenogeneic graft and the synthetic bone substitute. The common standard deviation was considered 0.01, which corresponds to 10% of the highest value for mean root resorption reported by Ru et al. [21]. The level of significance was set to 0.0125 to reproduce the measured root resorptions, and an associated effect size of 1.3538 was considered to reach 80% or more power in a one-way ANOVA model with three groups. The study design involved the use of seven rats per group (based on the type of cleft repair material used), including two animals for dropout, and a 16-week investigation period (Fig. 1).

Detailed information about the experimental setup was previously published [28–30]. All animal experiments were approved by the Governmental Animal Care and Use Committee (Reference No.: 81–02.04.2018.A342; Landesamt für Natur, Umwelt und Verbraucherschutz Recklinghausen, Nordrhein-Westfalen, Germany; dated: 11.01.2019) and were performed in accordance with the German animal welfare law (Tierschutzgesetz, TSchG) and European Union Directive 2010/63/EU. The study design also conformed with the Animal Research: Reporting of In Vivo Experiments (ARRIVE) Guidelines [31] and the Guide for the Care and Use of Laboratory Animals.

The animals (*N* = 21) were divided into three groups on the basis of the kind of grafting material to be used for alveolar-cleft repair: autologous bone from the ischial tuberosity of the hip, xenogeneic/human bone substitute (Maxgraft, Botiss Biomaterials, Krems, Austria) and synthetic β-TCP/HA bone substitute (Maxresorb, Botiss Biomaterials, Krems, Austria).

The artificial alveolar clefts were generated in the maxillary left side in 8-week-old male Wistar rats with an average weight of 465 ± 34 g. The cleft repair was performed 4 weeks later in a second surgery in the then 12-week-old animals with an average weight of 504 ± 36 g. After a consolidation phase of a further 4 weeks, the orthodontic appliance was inserted in the 16-week-old animals (average weight: 542 ± 32 g).

After 8 weeks of orthodontic tooth protraction, the rats were sacrificed under general anaesthesia through cervical dislocation, and the affected part of the maxilla was resected, including the corresponding tooth.

### Cleft creation and repair

The cleft preparation and cleft repair were done under general anaesthesia through the intraperitoneal (IP) injection of ketamine (80–100 mg/kg, Ketavet, Pfizer, Berlin, Germany) and medetomidine hydrochloride (0.15–0.25 mg/kg, Domitor, Orion Pharma, Espoo, Finland). Oxygen administration was guaranteed by endotracheal intubation, and anaesthetisation was achieved through the subcutaneous administration of buprenorphine (0.03–0.05 mg/kg, Temgesic, Indivior Limited, Berkshire, UK). Adjuvant antibiotic treatment was given using cefuroxime (15 mg/kg s.c., Fresenius, Bad Homburg, Germany) for 7 days. Afterwards, atipamezole hydrochloride (0.75 mg/kg, Antisedan, Orion Pharma, Espoo, Finland) was administered via IP to support the recovery process, and if applicable, buprenorphine (0.03–0.05 mg/kg) was given subcutaneously for a maximum of 5 days. After these interventions, the laboratory animals were replaced in their cages and were intensively monitored and observed until their full recovery.

The artificial cleft was created using an ultrasonic surgery device (Ø 1.7 mm, insert OT5, Mectron s.p.a., Carasco, Italy) for an osteotomy between the roots of the incisor and the first molar under irrigation with saline solution. Afterwards, bone wax (Bonewax, Ethicon, Johnson & Johnson Medical GmbH, Norderstedt, Germany) was used to preserve the artificial cleft. Finally, the wound was closed with a continuous resorbable suture (7/0 Vicryl, Ethicon, Johnson & Johnson Medical, Somerville, NJ, USA) [28].

**Fig. 2:**
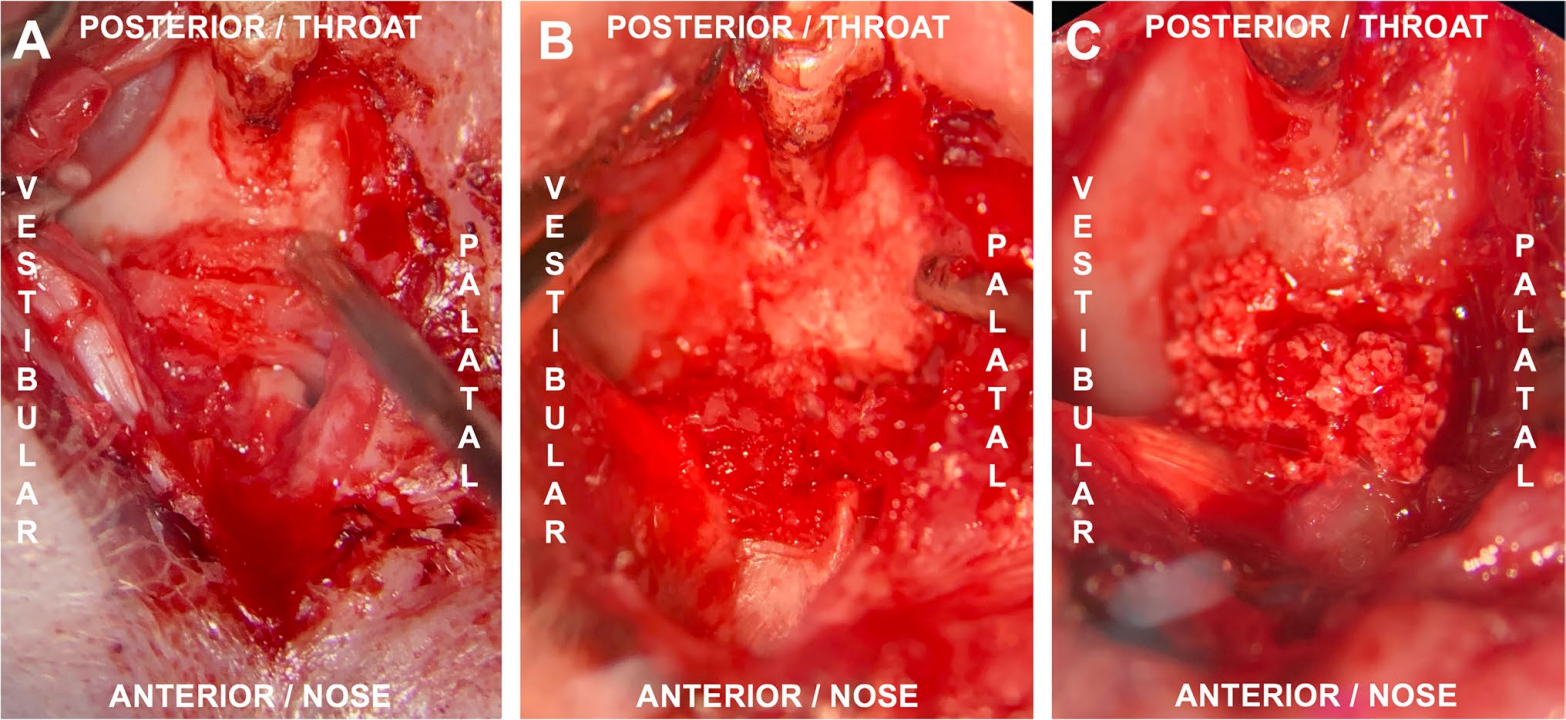
View of the operative situs of the left maxilla in the supine position: first molar above, mouth tip below: re-entry and cleft repair performed with **a** autologous bone from the ischial tuberosity of the hip, **b** xenogeneic/human bone substitute or **c** β-TCP/HA bone substitute material

For cleft repair, the soft tissue was deflected as in the previous operation. In the group with cleft repair using autografts, the bone was harvested before the cleft repair from the left hip’s ischial tuberosity [29]. Before the autologous bone or the other grafting materials (xenogeneic/human or synthetic substitute) were used, the cleft was prepared by removing the applied bone wax and refreshing the surrounding bone. Subsequently, maxillary reconstruction was achieved by applying the three different grafting materials (Fig. 2), and the autografts and human xenografts were sufficiently fixed via press-fit technique while the synthetic bone substitute material (β-TCP/HA) was carefully applied under condensation. Ensuing wound closure was done with continuous resorbable sutures (7/0 Vicryl, Ethicon, Johnson & Johnson Medical, Somerville, NJ, USA).

### Orthodontic tooth movement

**Fig. 3:**
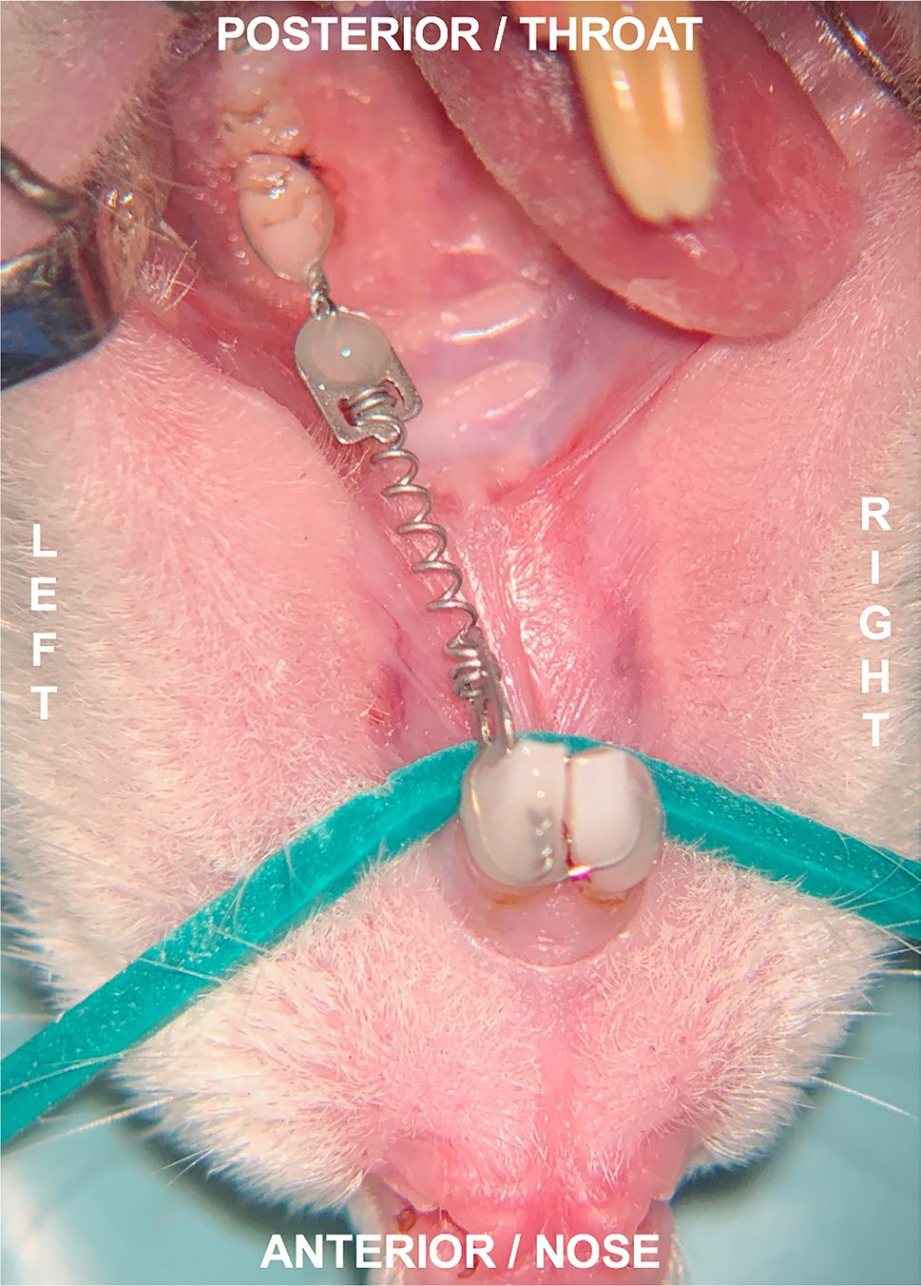
Rat in supine position: orthodontic appliance installed 4 weeks after cleft repair using a 0.14-N nickel–titanium closed-coil tension spring between the first molar and the incisors fixed with tension springs after conditioning of the teeth through acid etching using 39% phosphonic acid and a bonding agent and dental composite

After performing the anaesthesia and anaesthetic protocol as described for cleft creation and repair, a nickel–titanium closed-coil tension spring (33–54,495, PSM Medical Solutions GmbH, Gunningen, Germany) was applied between the incisors and the first upper-left molar, as in the study by Kirschneck et al. [32–34], to achieve a continuous force application of about 0.14 N [28]. The spring was fixed with wire ligatures (Ø 0.01″) and dental composite (Venus Flow, Kulzer GmbH, Hanau, Germany) via acid etching (Fig. 3).

### Microfocus computed tomography analysis

Radiological examination for determining tooth movement and root changes was carried out 2 days before (T0) and 8 days after (T1) placement of the orthodontic appliance using an in vivo microfocus computed tomography (µCT) system (U-CT OI, MILabs, Utrecht, the Netherlands) under a standardised setting with regard to general isoflurane anaesthesia and the radiological analysis protocol [28]. Additional radiological examinations were performed on days 15, 29, 43 and 57 (T2-T5).

The tooth movement distance was measured in the sagittal projections of µCT as in the study by Ru et al. Visible landmarks (i.e. the tips of the left first and second molars) were chosen to quantify the tooth movement. The distance was measured three times at each time point, and the measurements were averaged to obtain reliable results [22].

**Fig. 4:**
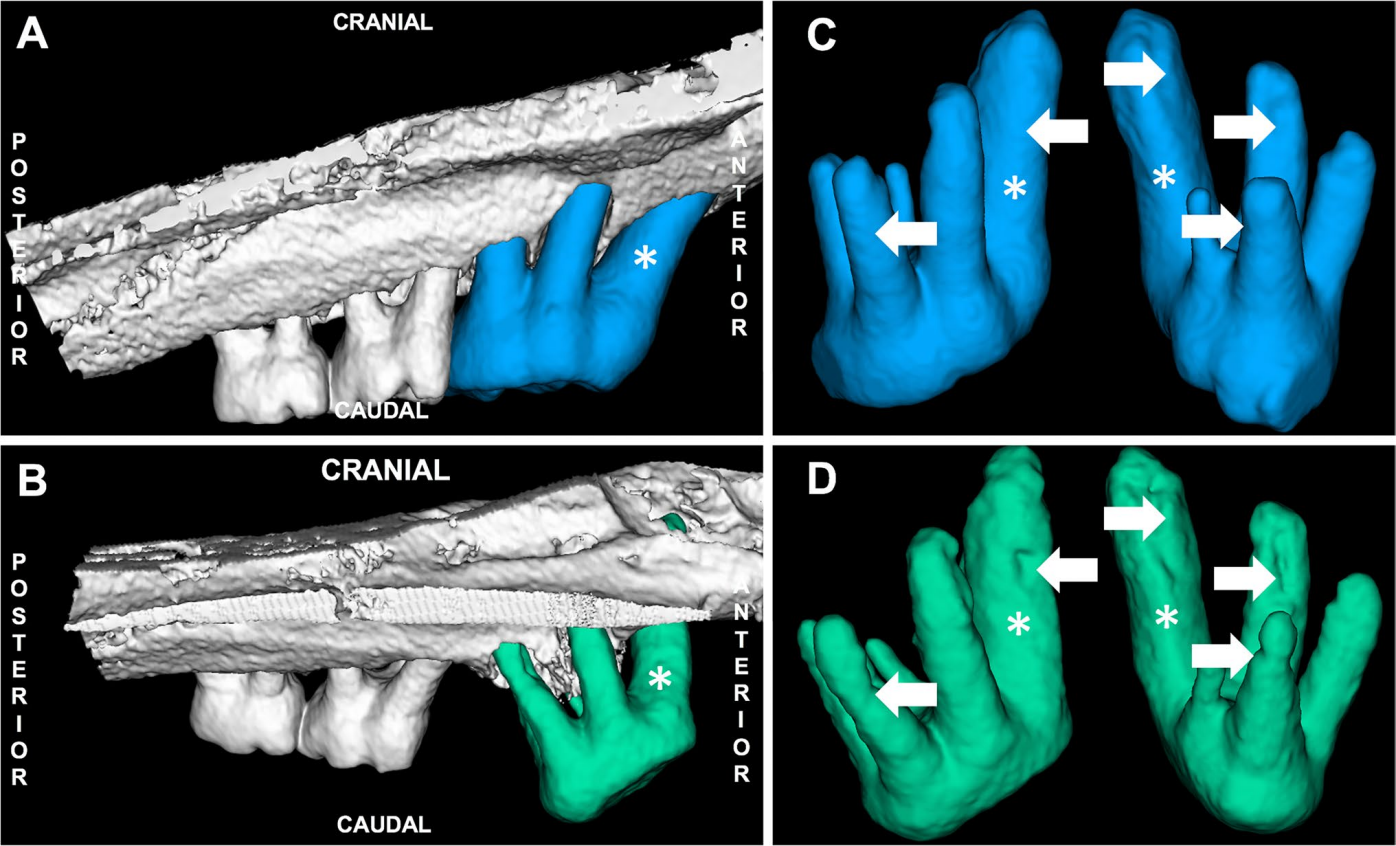
Radiologically determined root resorption of the first molar M1 (*). Locations of the teeth in the upper jawbone **a** before (in blue) and **b** after (in green) the orthodontic treatment. Comparison of the extracted teeth **c** before (in blue) and **d** after (in green) the orthodontic treatment with marked (white arrows) visible signs of root resorption

To analyse the changes in the roots with regard to possible signs of resorption, the first molars were segmented in μCT images using all the anatomical planes (Fig. 4). All the roots were delineated separately, and their volumes at each time point were computed. The root resorption was calculated for each root by subtracting the root volume at each time point for every animal from the root volume at T0.

### Histomorphometric analysis

**Fig. 5:**
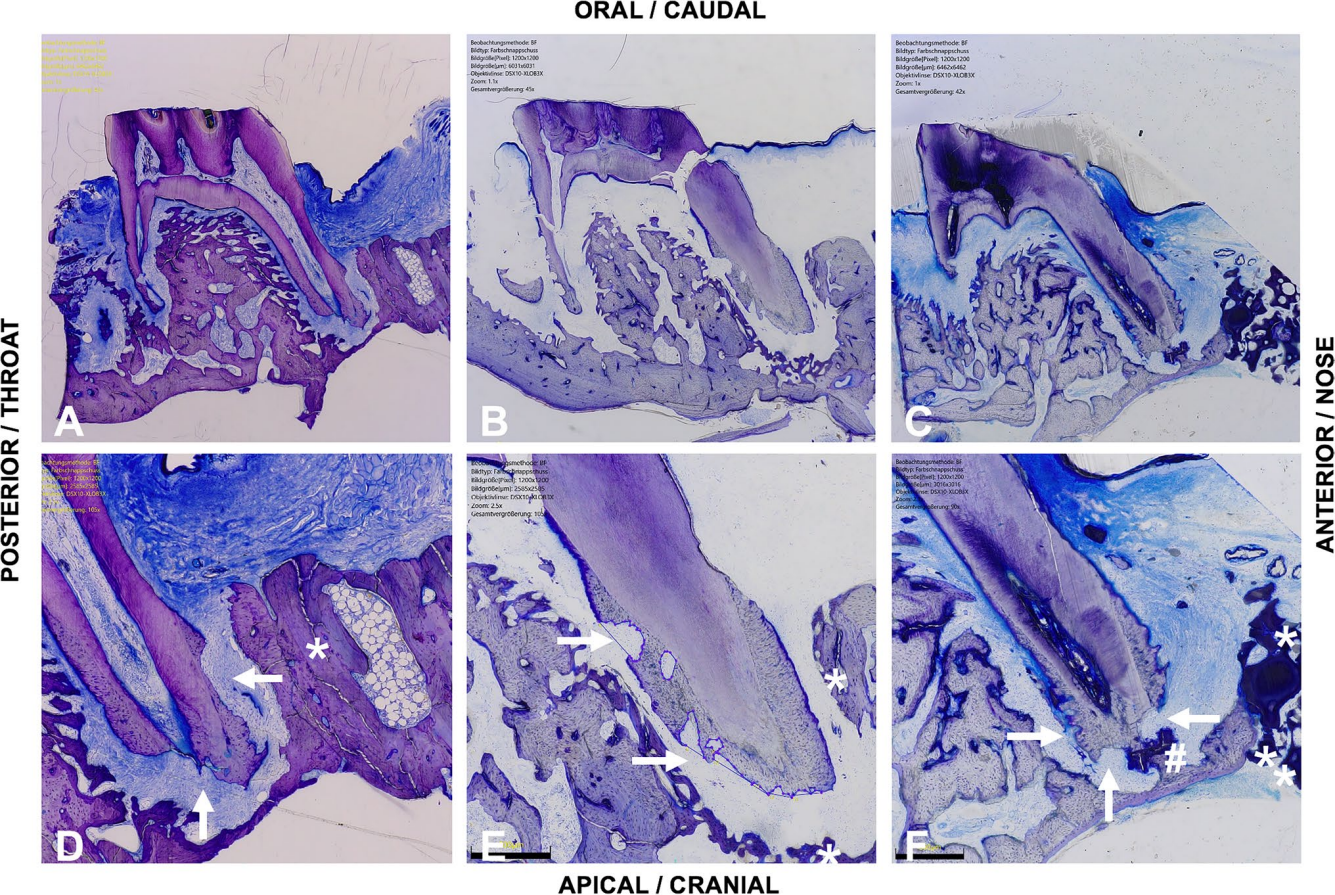
Histological longitudinal section (toluidine blue stains) through the tooth and surrounding hard- and soft-tissue Sect. 84 days after cleft repair using **a**, **d** autologous bone, **b**, **e** xenogeneic/human bone and **c**, **f** synthetic bone substitute (beta-tricalcium phosphate/hydroxyapatite): overview: **a**–**c**, 100 × magnification; detailed view: **d**–**f** up to 350 × magnification; root resorption (arrows), persistent bone/substitute (*) and signs of ankylosis (#)

The preparation of the histomorphometric samples followed an established procedure [30]. The samples were stored in 4% formalin, decalcified in ethylenediaminetetraacetic (EDTA) and shock frozen in liquid nitrogen. Afterwards, they were embedded and cut into 5- to 7-μm-thick longitudinal sections through the tooth and the surrounding hard and soft tissue. The samples were then fixed in acetone for 10 min and stained with toluidine blue according to the routine protocols. Evaluation was carried out using a light microscope with software support (Olympus digital microscope DSX-1000, Olympus Hamburg, Germany) (Fig. 5a–c).

A total of 19 slices, six in the autologous-bone and xenogeneic/human-bone group and seven in the synthetic-bone-substitute group, were conducted and evaluated by one investigator. The mesial root of the tooth was defined as general region of interest. The evaluation was carried out using a light microscopy with software support (Olympus digital microscope DSX-1000, Olympus Hamburg, Germany) (Fig. 5a–c). The samples were investigated under qualitative aspects regarding signs of inflammatory reactions, necroses and ankylosis as well as the number of multinucleated giant cells. Furthermore, the dimensions of resorption lacunae (mm^2^) were determined as the area between the intact parts of the root surfaces (Fig. 5d–f). If several lacunae occurred, their individual areas were added to the total resorption.

## Results

Neither wound healing disorders nor acute inflammatory processes were observed after both surgical interventions.

However, even though wound healing was good after the surgical procedures, two animals died after the second operation due to inhalation problems or circulatory failure [28]. As such, there were now only six animals each in the autologous-bone and xenogeneic/human-bone groups and seven in the synthetic-bone-substitute group. In all, 11 broken orthodontic appliances were found during the radiological follow-up imaging period, and among these, one apparatus came loose twice.

### Tooth movement

**Fig. 6:**
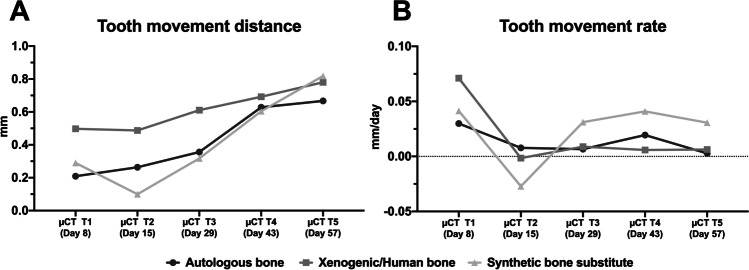
Quantification of tooth movement depending on the grafting materials used for cleft repair in microfocus computed tomography over a period of 57 days or 8 weeks, respectively: **a** distance of tooth movement and **b** tooth movement per week

After an initial period of 7 days (µCT T1), the mean tooth movement of the first molar was 0.21 ± 0.08 mm in the autologous-bone group, 0.50 ± 0.54 mm in the xenogeneic/human-bone group and 0.29 ± 0.12 mm in the synthetic-bone-substitute group (Fig. 6a). After 8 weeks (µCT T8), a distance of 0.82 ± 0.72 mm was measured in the synthetic-bone-substitute group, 0.78 ± 0.69 mm in the xenogeneic/human-bone group and 0.67 ± 0.27 mm in the autologous-bone group. The corresponding mean tooth movement rate range during the orthodontic movement was from 0.003 ± 0.006 (T5) to 0.03 ± 0.011 mm/day (T1) in the autologous-bone group, from − 0.002 ± 0.036 (T2) to 0.071 ± 0.077 mm/day (T1) in the xenogeneic/human-bone group and from − 0.027 ± 0.027 (T2) of 0.41 ± 0.017 mm/day (T1) in the synthetic-bone-substitute group (Fig. 6b). With regard to the amount of tooth movement, no statistically significant difference was found among the groups (autologous-bone group: *p* = 0.52; xenogeneic/human-bone group: *p* = 0.87; synthetic-bone-substitute group: *p* = 0.29) on all the measurement days (e.g. T1: *p* ≥ 0.58; T5: *p* ≥ 0.85) and between the measurement times (T1 vs. T5) within each group.

Thus, varying degrees of tooth movement took place over the entire period, characterised by a high initial tooth movement (1st week) followed by a relapse (2nd week, especially in the synthetic-bone-substitute group) and a subsequent slower forced tooth movement (3rd to 8th weeks).

### Microfocus computed tomography imaging

**Fig. 7:**
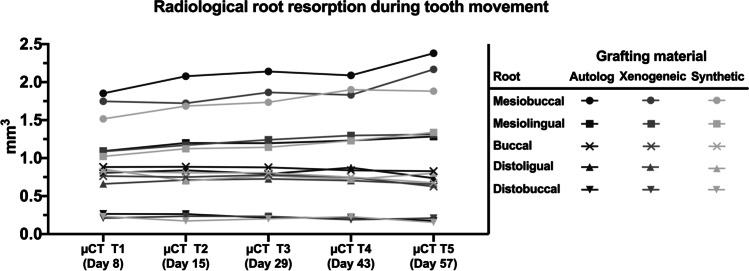
Radiologically measured progression of all the first-molar root resorptions over the 8-week tooth movement period depending on the used bone substitutes for jaw reconstruction

Radiological detectable resorptions occurred to varying extents on all the roots. The occurrence of resorption increased with higher proximity to the filled cleft defect. Consequently, the largest resorption phenomenon occurred at the mesiobuccal root, and the smallest, at the distobuccal root. During orthodontic tooth movement, no significant difference in root resorption was observed between the beginning (µCT T1) and the end (µCT T5) of tooth protraction (Fig. 7).

Although resorption progression occurred in all the three groups for the mesiobuccal root during the orthodontic tooth movement (µCT T1 vs. µCT T5: autologous-bone group 1.85 ± 0.39 mm^3^ vs. 2.38 ± 0.35 mm^3^, *p* = 0.30; xenogeneic/human-bone group 1.75 ± 0.45 mm^3^ vs. 2.17 ± 0.26 mm^3^, *p* = 0.54; synthetic-bone-substitute group 1.52 ± 0.42 mm^3^ vs. 1.88 ± 0.41 mm^3^, *p* = 0.60) and between the autologous-bone and synthetic-bone-substitute groups at the beginning and end of this intervention (autologous-bone group vs. synthetic-bone-substitute group: µCT T1 1.85 ± 0.39 mm^3^ vs. 1.52 ± 0.42 mm^3^, *p* = 0.41; µCT T5: 2.38 ± 0.35 mm^3^ vs. 1.88 ± 0.41 mm^3^, *p* = 0.14), these differences were not statistically significant.

With regard to the other roots, no progressive changes in root resorption were observed regardless of the grafting material used, except in the mesiolingual root, which appeared to be minor (µCT T1 vs. µCT T5: autologous-bone group 1.09 ± 0.19 mm^3^ vs. 1.29 ± 0.15 mm^3^, *p* = 0.76; xenogeneic/human-bone group 1.09 ± 0.38 mm^3^ vs. 1.31 ± 0.37 mm^3^, *p* = 0.63; synthetic-bone-substitute group 1.02 ± 0.07 mm^3^ vs. 1.34 ± 0.24 mm^3^, *p* = 0.23).

As for the influence of the grafting material, the most affected mesiobuccal root showed the greatest damage at all the time points in the autologous-bone group, followed by the xenogeneic/human-bone and synthetic-bone-substitute groups (Table 1). Thus, the root resorptions in the autologous-bone group ranged from 1.85 ± 0.39 mm^3^ (µCT T1) to 2.14 ± 0.86 mm^3^ (µCT T5); those in the xenogeneic/human-bone group, from 1.72 ± 0.45 mm^3^ (µCT T1) to 2.17 ± 0.26 mm^3^ (µCT T5); and those in the synthetic-bone-substitute group, from 1.52 ± 0.42 mm^3^ (µCT T1) to 1.90 ± 0.52 mm^3^ (µCT T4).

**Table 1:** Radiologically determined root resorption on all the five roots depending on the grafting material used over the 8-week treatment period

µCT	Grafting material	Radiological root resorption (mm^3^)									
		Mesiobuccal		Mesiolingual		Buccal		Distolingual		Distobuccal	
		Mean	SD	Mean	SD	Mean	SD	Mean	SD	Mean	SD
T1 (day 8)	Autograft	1.85	0.39	1.09	0.19	0.88	0.22	0.81	0.11	0.27	0.05
	Human xenograft	1.75	0.45	1.09	0.38	0.76	0.31	0.66	0.2	0.21	0.05
	β-TCP/HA substitute	1.52	0.42	1.02	0.07	0.82	0.16	0.85	0.2	0.23	0.09
T2 (day 15)	Autograft	2.08	0.59	1.2	0.35	0.89	0.21	0.84	0.12	0.26	0.07
	Human xenograft	1.72	0.68	1.17	0.4	0.75	0.22	0.71	0.16	0.24	0.1
	β-TCP/HA substitute	1.68	0.19	1.12	0.14	0.81	0.01	0.7	0.04	0.17	0.09
T3 (day 29)	Autograft	2.14	0.86	1.2	0.38	0.88	0.33	0.79	0.21	0.21	0.08
	Human xenograft	1.86	0.42	1.24	0.24	0.78	0.28	0.73	0.19	0.23	0.07
	β-TCP/HA substitute	1.73	0.33	1.14	0.12	0.81	0.08	0.77	0.13	0.2	0.09
T4 (day 43)	Autograft	2.09	0.29	1.23	0.32	0.84	0.26	0.87	0.3	0.22	0.09
	Human xenograft	1.83	0.38	1.3	0.36	0.75	0.34	0.7	0.22	0.19	0.1
	β-TCP/HA substitute	1.9	0.52	1.23	0.18	0.75	0.25	0.72	0.16	0.23	0.13
T5 (day 57)	Autograft	2.38	0.35	1.29	0.15	0.83	0.17	0.74	0.19	0.18	0.04
	Human xenograft	2.17	0.26	1.31	0.37	0.63	0.24	0.66	0.19	0.21	0.07
	β-TCP/HA substitute	1.88	0.41	1.34	0.24	0.67	0.16	0.8	0.18	0.15	0.1

### Histomorphology analysis

In general, no signs of inflammatory reactions, increased number of multinucleated giant cells, necrosis and ankylosis were found. Only one xenogeneic/human-bone sample demonstrated an inflammatory reaction of the apical root, while in the autologous-bone and the synthetic-bone-substitute group one sample each suggested an ankylosis of the mesial root (Fig. 5f).

**Fig. 8:**
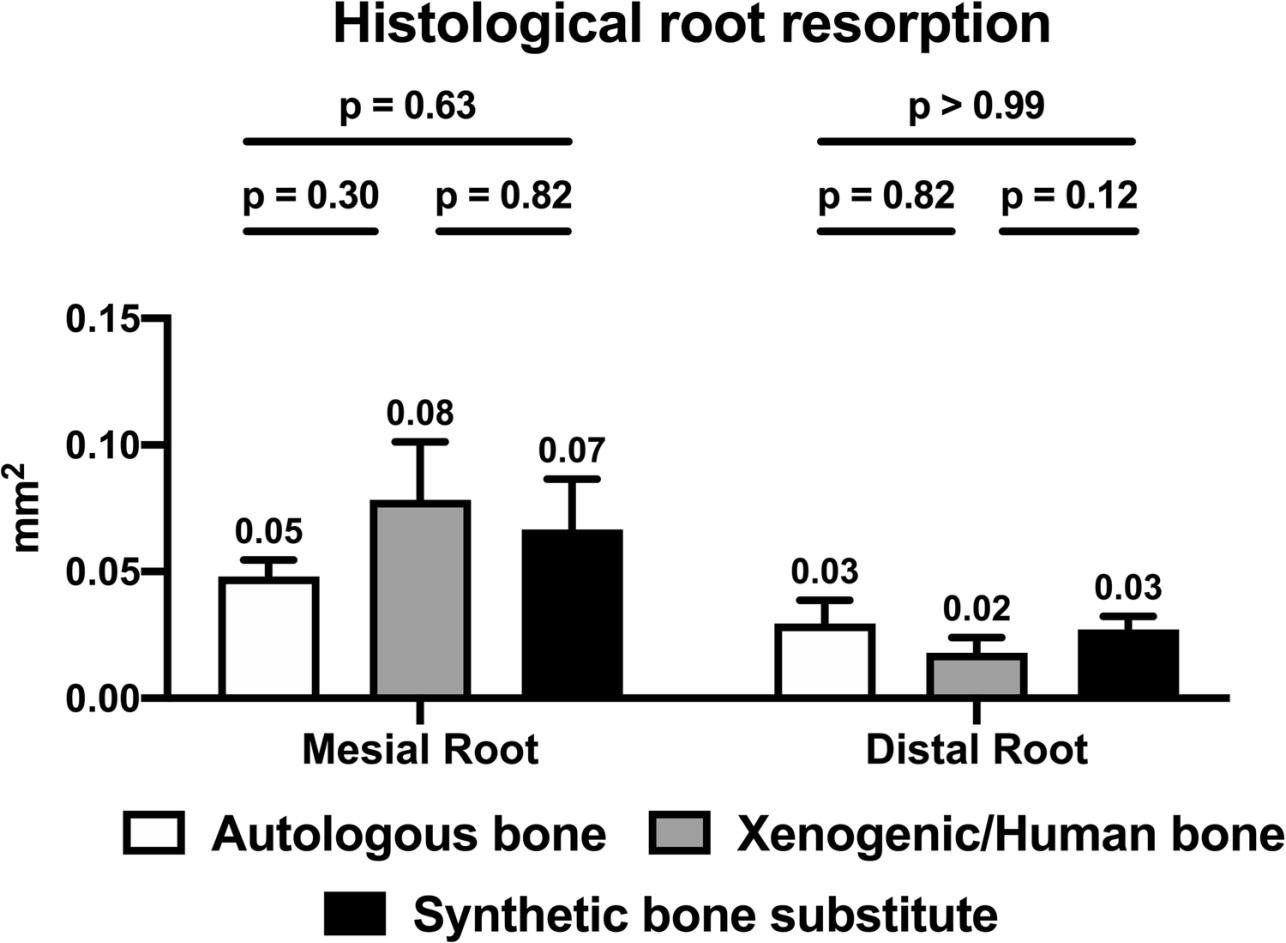
Histological analysis of the tooth samples for root resorption after the completion of the orthodontic movement phase (day 57): bar chart of the mean values and the corresponding *p* values for comparisons between the different groups

In the histological longitudinal section, resorption lacunae of varying degrees were found for both the mesial and distal roots (Fig. 8), whereby mainly the mesial root close to the repaired cleft was affected. The highest extent of resorption was found in the xenogeneic/human-bone group (0.078 ± 0.056 mm^2^), followed by the synthetic-bone-substitute group (0.067 ± 0.049 mm^2^) and the autologous-bone group (0.048 ± 0.015 mm^2^). However, these differences were not statistically significant.

Furthermore, differences in the amount of resorption were found between the mesial and distal roots, but these were statistically significant only in the xenogeneic/human-bone group (mesial: 0.078 ± 0.056 mm^2^ vs. distal: 0.018 ± 0.015 mm^2^; *p* = 0.01).

## Discussion

Alveolar clefts are unique with regard to the special oral and nasal mucosa lining in the defect region. However, the animal models previously used in rodents were not in accordance with these differing cleft morphologies [14–22]. Additionally, subsequent and equivalent orthodontic tooth movement in the cleft repair region of these models is not possible because the defect is too far away from the molar or the alveolar cleft is not completely interrupting the continuity of the bone. Therefore, to prevent this methodical deficit, the present study design was established, which makes use of grafting materials similar to human alveolar clefts and enables sufficient cleft repair with autografts from the ischial tuberosity of the hip as well as subsequent orthodontic tooth movement [28, 29].

Autologous bone especially from the iliac crest remains the gold standard [28] for packing the bony alveolar defect [6–8]. It has some weaknesses, however, including limited bone supply, demand for an additional donor site, related postoperative morbidity (pain, haematoma and delayed ambulation) and inherent susceptibility to resorption in the long term [6–10]. For this reason, different tissue-engineered bone substitutes have been investigated, which should improve bone fusion and eliminate donor site morbidity [8, 35–37].

Calcium phosphate ceramics are among the candidate bone substitute grafting materials contained in various types of alloplastic biomaterials, such as calcium sulphate, TCP or β-TCP [37, 38]. To ensure the ideal resorption of the material, the preferred ratio of HA and β-TCP ranges from 65:35 to 55:45 [39, 40]. In this context, it was reported that the healing process of β-TCP seems similar to that of the autogenous bone graft from the iliac crest at least in a goat model [41]. Additionally, de Ruiter et al. found increased bone formation in the β-TCP group compared to the autograft group (22.90 ± 5.62% vs. 20.87 ± 5.40%), but the increase was not statistically significant [41].

Human allografts are alternatives to synthetic bone substitutes and are already being used clinically in cleft repair. In particular, the use of allogeneic bone grafts customised through computer-aided design/computer-aided manufacturing is gaining in popularity and seems to enable complete osseous integration and fusion of the grafts in the recipient site [26, 27]. However, it must be mentioned that animal studies are of limited value in this context because human bone allografts in animals must be assessed as xenogeneic transplants that may lead to immunological reactions of the collagen structures [42].

A sufficient analysis of bone and root changes with regard to graft healing and root resorption can be done in animal research using μCT [43–45]. Due to the isotropic voxel sizes and standardised voxel units, the volumetric μCT data are appropriate for quantitative analysis using high-resolution 3D imaging in in vivo and ex vivo laboratory settings [43, 45–49].

Ru et al. have investigated real-time root resorption in graft sites in rats during orthodontic tooth movement after alveolar-defect packing with different bone substitutes, using μCT [21, 22, 45]. The defect repair was performed optionally with natural bovine cancellous bone particles or a synthetic bone substitute based on a mixture of 60% HA and 40% β-TCP, while in the control group no graft was used after maxillary-first-molar extraction [21]. The least amount of tooth movement and the lowest root resorption and crater volumes were detected in the synthetic-bone-substitute group. In general, the highest root resorptions were found in the apical region of the mesiobuccal roots in all the groups [22]. Finally, the authors concluded that the β-TCP/HA substitute has better osteoconductive potential and induces less root resorption compared to bovine grafting and the naturally recovered defect sites.

Similar results for the β-TCP/HA bone substitute were found in the present study, but the findings of this study cannot be directly compared with those of Ru et al. as the defect models that were used and the kind of μCT analysis that was conducted in the two studies were different [21]. Ru et al. investigated different areas of the roots with regard to the incidence of signs of resorption [22] whereas, in the present investigation, the overall resorption for each root was analysed. In general, the radiological examination in the present study also revealed that resorptions occurred in all the roots in the course of tooth movement. In concordance to the findings of Ru et al., in the present study the largest resorptions were found on the mesiobuccal root close to the repaired cleft. Apart from the fact that the foregoing might have been because this root is the largest, it seems that this root carries an increased risk for undesirable alterations because it was the only root that showed a progression of resorption during the tooth movement. However, the increase here was not statistically significant. In contrast, in the present study, no progression was observed on the four other roots. In fact, the observed resorptions differed at first glance between the individual groups (autologous-bone group > xenogeneic/human-bone group > synthetic-bone-substitute group). However, the differences were not statistically significant, and the changes could not have been only caused by the grafting materials as the roots had no contact to these in the beginning. Nevertheless, as the changes on the mesial root increased compared to the other roots during the orthodontic movement, the bone substitutes must have had proportional effects. The histological analysis in the present study demonstrated the most severe root damage in the xenogeneic/human-bone group, followed by the autologous-bone and synthetic-bone-substitute groups, but the differences among the groups were also not statistically significant.

In this context, Seifi and Ghoraishian reported that when they used human bone as a xenograft for cleft repair in canines, the tooth movement showed both decreased root resorption for the teeth in the allogeneic bone and a significant increase in tooth movement velocity compared with the untreated control site [50].

In contrast, Ru et al. reported intermittent tooth movement velocity in all the groups in their study (β-TCP/HA substitute, bovine xenograft or unpacked). The velocity was significantly higher in the 1st and 3rd weeks than in the 2nd and 4th weeks, respectively [22]. They observed the greatest tooth movement in the control group, which was approximately 0.35 mm after 14 days, followed by the xenogeneic/bovine-bone group (0.3 mm) and the β-TCP group (0.25 mm) [21]. The corresponding distances after 28 days were approximately 0.98 mm, 0.86 mm and 0.83 mm, respectively [22]. In this context, Kirschneck et al. reported 0.8 ± 0.2 mm tooth movement in an uncompromised jaw 14 days after the mesial tipping, with a mesial-root torque of 0.4 ± 0.3 mm, 0.9 ± 0.2 mm tooth movement and 0.4 ± 0.3 mm mesial-root torque after 28 days [34].

The mean distance of orthodontic tooth movement in the present study after 14 days ranged from 0.26 ± 0.26 mm in the autologous-bone group to 0.50 ± 0.70 mm in the xenogeneic/human-bone group, and that after 28 days ranged from 0.32 ± 0.15 mm in the synthetic-bone-substitute group to 0.61 ± 1.04 mm in the xenogeneic/human-bone group. After the 8th week of tooth movement, the largest distance was about 0.82 ± 0.72 mm in the synthetic-bone-substitute group, followed by 0.78 ± 0.69 mm in the xenogeneic/human-bone group and 0.67 ± 0.27 mm in the autologous-bone group. Thus, the initial tooth movement velocity in the present study appears to be comparable to that in the study by Ru et al., but the total movement time was significantly slower. Furthermore, the tooth movement was also slower than that in the study by Kirschneck et al. [34]. Therefore, it seems that further tooth movement is inhibited by the grafting material used.

In summary, the differences in root resorption and tooth movement between the bone graft substitutes, as well as autologous bone as gold standard, were not statistically significant at any time. An interpretation of the differences is more of speculative nature. The materials used in this study for cleft repair seem to have a similar effect on orthodontic tooth movement and the development of root resorptions. It is important to verify these findings in further basic research.

## Conclusion

With regard to the limitations of the animal study design, it appears that regardless of the grafting material used, tooth movement slowed down and the root resorption increased in the present study. This occurs mainly at the mesial root, which was the closest to the repaired cleft. Neither radiologically nor histologically statistically significant differences were found between root resorptions regardless of the bone substitute used for jaw reconstruction. Furthermore, the same distance of tooth movement was detected during the research period. Therefore, the development of root resorptions should have a secondary role in choosing a suitable grafting material for cleft repair.

## Supplementary Information

Below is the link to the electronic supplementary material.

Supplementary file1 (PDF 615 KB)

Supplementary file2 (PDF 94 KB)
