# Supplementary material for: Development of root resorption during orthodontic tooth movement after cleft repair using different grafting materials in rats
Source: Clin Oral Investig. 2022 May 14;26(9):5809–21. doi: 10.1007/s00784-022-04537-3 (PMC9474460; doi:10.1007/s00784-022-04537-3)
Supplement: Supplementary file 2 — Supplementary file2 (PDF 94 KB) [file 784_2022_4537_MOESM2_ESM.pdf]

Alfred- Herrhausen- Str. 44
D-58455 Witten

Telefon: 02302/ 926-645
Telefax: 02302/ 926-661
Lehrstuhl für Kieferorthopädie
- Lehrstuhlinhaber -

Prof. Dr. med. dent. Gholamreza Danesh
Gholamreza.Danesh@uni-wh.de

Universität Witten/Herdecke, Alfred-Herrhausen-Str. 44, 58455 Witten

Editorial Office
Scientific Reports

20.01.2022

Dear Professor Richard White,
Dear Professor Daniel McNaally

First of all, we would like to thank you and the reviewer for the helpful comments, which further developed and improved our manuscript entitled "Comparison of soft tissue simulations between two planning software programs for orthognathic surgery". We have made modifications and thank you to offer us the opportunity to resubmit our manuscript.
The concerns of the reviewers were responded on a point-by-point basis and all changes in the manuscript were coloured.
In accordance with the instructions for authors, the manuscript has been approved by all authors and all of them have taken due care to ensure the integrity of their work.
Thank you and your editorial board for your efforts.

Respectfully,

Stephan Christian Möhlhenrich, DMD, MSc, MBA, PhD

Private Universität Witten/Herdecke gGmbH ·
Geschäftsführung: Prof. Dr. med. Martin Butzlaff (Präsident), Dipl. oec. Jan Peter Nonnenkamp (Kanzler).·
Sitz der Gesellschaft: Witten · Amtsgericht Bochum HRB 8671 · Steuer-Nr.: 348 / 5866 / 0314· Ust-IdNr.: DE 126 880 235 ·
GLS Gemeinschaftsbank eG Bochum, Konto 353 500, BLZ 430 609 67, IBAN :DE76 4306 0967 0000 3535 00, BIC: GENODEM1GLS ·
Deutsche Bank AG Witten, Konto 8 327 777, BLZ 430 700 61, IBAN: DE94 4307 0061 0832 7777 00, BIC: DEUTDEDE430 ·
Sparkasse Witten, Konto 10 900, BLZ 452 500 35, IBAN: DE55 4525 0035 0000 0109 00, BIC: WELADED1WTN
